# *Salmonella* Typhimurium Veterinary Clinic Outbreak

**DOI:** 10.3201/eid1112.041295

**Published:** 2005-12

**Authors:** John F. Prescott

**Affiliations:** * University of Guelph, Guelph, Ontario, Canada

**Keywords:** Salmonella, letter, veterinary

**To the Editor:** The Emerging Infectious Diseases 2004 issue on zoonotic diseases (volume 10, number 12) included a careful and comprehensive description of a *Salmonella enterica* serovar Typhimurium outbreak associated with a veterinary clinic in New York (*1*). In the outbreak, 2 cats and 1 dog had dental procedures performed, and the 3 owners, 2 clinic technicians, and a friend of an affected owner all contracted salmonellosis caused by the same strain. An isolate was obtained from an animal, but a source for the *Salmonella* outbreak was not identified.

I get 1 or 2 phone calls each year from veterinarians in Canada regarding recurrent problems of salmonellosis in their clinics, though rarely with human infections. The advice I give the veterinarians, which stops the problem, is to stop using clindamycin as a routine prophylactic agent when carrying out dental procedures. The marked disruption of the colonic anaerobic microflora by oral clindamycin will reduce the number of *Salmonella* organisms required to establish infection to very few. In veterinary journals, advertising for clindamycin focuses on its use in prophylaxis of infections after dental procures such as cleaning, scaling, and extractions. Veterinary practitioners typically respond to my advice with initial disbelief because it challenges use of a procedure that is seen as standard in veterinary practice.

That "all 3 animal patients were treated after the [dental] procedure with a prophylactic course of clindamycin" is the most meaningful factor in this outbreak, but this point was not commented on by the authors. The apparently increasing use in North American dogs and cats of biologically appropriate raw foods diets, in other words raw meat, may be exacerbating the problem since most such diets are contaminated with *Salmonella* spp (*2*). In addition, *Clostridium difficile* infection is increasingly recognized as a common cause of diarrhea in dogs (*3*) and might also develop in some animals treated with clindamycin, just as it does in humans.

A number of antimicrobial drugs are likely to be as effective as clindamycin for dental prophylaxis, if indeed any antimicrobial drug is truly needed, and these are considerably less likely to produce what is probably the side effect described in this report. Moreover, a canine dentistry text states, "Most routine dental cleaning procedures do not require antibiotic administration. The American Dental Association, the American Academy of Oral Medicine, and the Council on Scientific Affairs advise against the routine use of antibiotics for dental cleaning procedures" (*4*). The case reported by Cherry et al. probably supports this recommendation.
